# Thyroid Cancer: Current Molecular Perspectives

**DOI:** 10.1155/2010/351679

**Published:** 2010-03-29

**Authors:** Francesca Giusti, Alberto Falchetti, Francesco Franceschelli, Francesca Marini, Annalisa Tanini, Maria Luisa Brandi

**Affiliations:** Regional Centre for Hereditary Endocrine Tumors, Unit of Metabolic Bone Diseases, Department of Internal Medicine, University of Florence, Viale Morgagni 85, 50135 Florence, Italy

## Abstract

The thyroid cancer is a rare oncological entity, representing no more than 1% of all human malignant neoplasms. Recently, it has been demonstrated a sharp increase in incidence of differentiated thyroid carcinoma, equally occurring in both sexes. So far, multiple genetic alterations have been identified in differentiated thyroid carcinoma, leading to investigate the clinical utility of genetic studies. In particular, molecular genetic approaches searching for gene mutations in the material collected by fine needle ago-biopsy may have a particular utility in small nodules and in those specimens with an indeterminate cytology. The expansion of knowledge about genetic mutations occurring in different thyroid tumors has characterized recent years, allowing the identification of a correlation between specific mutations and phenotypic characteristics of thyroid cancers, essential for their prognosis. This review will briefly report on the histological features and the new entity represented by thyroid microcarcinoma and will focus on both environmental and genetic aspects associated with the occurrence of thyroid cancer.

## 1. Introduction

The thyroid cancer (TC) is a rare oncological entity, representing no more than 1% of all human malignant neoplasms. However, it represents the most common malignant endocrine neoplasia whose incidence has progressively increased over the past two decades, according to the majority of Tumors Registries. In fact, the data collected at the Surveillance Epidemiology and End-Results Cancer Registries program (SEER) [1] indicate an annual average prevalence of thyroid carcinoma (TCa) of 6.6/100.000 (9.5 and 3.5/100.000 for women and men, resp.) with an annual increase >5% in the period 1975–2002.

In a very recent study in the United States (U.S.), a sharp increase in incidence of differentiated TC, equally occurring in both sexes and independently by the sizes of the lesions, has been reported. However, the detection at a preclinical stage, through neck ultrasounds (US), of a greater number of asymptomatic TCas and the progressive increase in both the prevalence and incidence of nodular thyroid disease cannot represent the only explanation for these increased incidence rates. Such findings are mainly due to an improved diagnostic histopathology with particular regard to the cytological diagnosis on fine needle ago-biopsy (FNAB). Consequently, other factors such as environmental influences and molecular alterations must be taken into account [2].

This review will briefly report on histological features and the new entity represented by thyroid microcarcinoma (TmCa) and will focus on both environmental and genetic aspects associated with the occurrence of TC.

## 2. Histological Features

TCs are divided into papillary carcinoma (PTC) (MIM #188550), follicular carcinoma (FTC) (MIM #188470), medullary thyroid carcinoma (MTC) (MIM #155240), anaplastic thyroid carcinoma (ATC), primary lymphoma of the thyroid (PLT), and primary sarcoma of the thyroid (PST).

**Figure 1:**
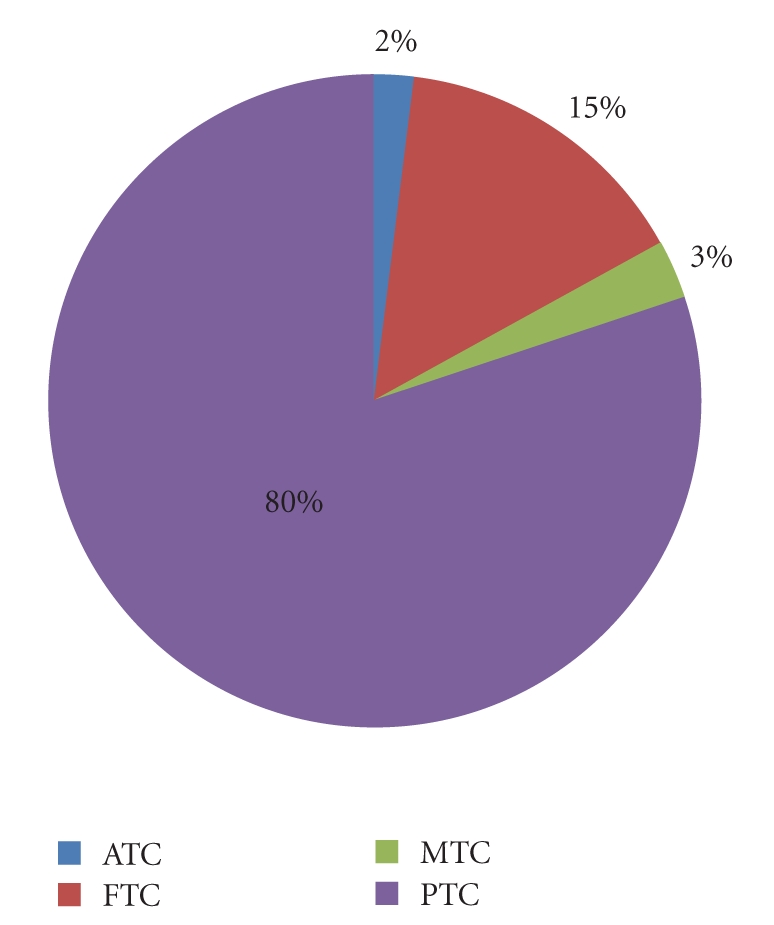
Frequency of thyroid neoplasms.

The PTC accounts for 80% of all thyroid malignancies [3] whereas FTC, the second most common malignancy of TC, represents approximately 15% of cases [4]. The MTC represents 3% of thyroid malignancies [4]. ATC, approximately representing 2%, is the most aggressive form of TC, while PLT and PST are very rare (Figure 1).

Through a careful revision of several published studies, a correlation between age of incidence and histological type can be established. In fact, PTC is more frequent in childhood and <50 years [5], FTC in patients <60 years [6], and the ATC 60–70 years [7] (Table 1).

## 3. Thyroid Microcarcinoma (TmCa): A “New Entity”

TmCa, diameter <1 cm, is an increasing pathological finding that could be regarded as a thyroid incidentaloma. In fact, as it happens for adrenal gland incidentaloma, TmCa is occasionally identified at US of the neck performed for other reasons. Most of TmCas are PTC with a sclerotic appearance of the nodule and similar prevalence in both sexes. TmCa is rare in children; whereas in autoptic series of adults, their frequency is similar in all of the age groups, indicating that in the absence of screening techniques enabling their identification TmCas occur in young adults and most cases regress or do not ever reach the clinical expression [8].

## 4. Mortality

Despite the significant increase in its incidence, mortality for TC has not increased in equal measure. It appears two times higher in female subjects with a rate of the annual mortality between 0.4–2.8 and 0.2–1.2/100.000, respectively, for women and men [1].

## 5. Risk Factors

The multifactor etiology of TC is the result of complex genetic and environmental factors interaction in individuals at risk. Epidemiological studies suggest the following main risk factors.

## 6. Gender and Age

TC is 2–4 times more frequent in women who generally exhibit a better prognosis than men in whom a higher malignant progression of nodules has been reported. It is rare in patients aged <16 years, presenting an annual incidence of 0.02–0.3/100.000, and extremely uncommon below the age of 10 years [9–11]. Its incidence increases with ageing and the average age at diagnosis is 45–50 years. However, the following issues have to be stressed: (1) although rare, the presence of TC in childhood accounts for a more advanced disease at diagnosis; and (2) in patients aged >60 years an increased risk for malignancy of thyroid nodules has been observed.

## 7. Ethnic Differences

A geographic and ethnic variability of TC incidence has been reported. In areas such as Iceland, Hawaii, the Philippines, Japan, and Israel its incidence is higher than in North America, Canada, and U.S.

In U.S., the TCa is more frequent in Caucasian descent subjects than Afro-American, Hispanic, Hawaiian, Chinese, or Japanese women, whose incidence is still twice as high in their countries of origin. All these findings suggest that such differences may be attributable to both environmental (e.g., dietary habits) and genetic factors [9, 12].

## 8. Environmental Factors

In this section we will consider the role of several parameters such as the (1) exposure to ionizing radiations, (2) age at the time of the exposure, (3) presence of a previous history of benign thyroid disease, (4) role of the dietary iodine intake, (5) role of the body mass index, and (6) role of hormonal factors.

### 8.1. Previous Exposure to Ionizing Radiation

The role of a previous exposure to ionizing radiation in thyroid carcinogenesis has been established since 1950 following the explosion of the atomic bomb in Japan. 

Previous exposure to ionizing radiation for external irradiation of the neck increases the incidence of thyroid nodules, either benign or malignant, and palpable nodules are detected in 20%–30% of people exposed to radiation [13], as well it happens in pediatric patients undergoing radiation therapy for oncological and haematological malignancies such as lymphoma or leukemia [14, 15].

The minimum latency period between exposure and clinical evidence of thyroid disease has been reported to be at least 4-5 years, reaching the maximum peak 20 years from exposure to decrease thereafter. The risk increases from medium doses above 10 cGy, and for doses up to 1500 cGy a linear dose-cancer risk can be observed. For higher doses the risk decreases probably in relation to radio-induced cell necrosis.

### 8.2. Age at the Time of Irradiation

It represents the main risk factor and after 15–20 years there is no longer an increased risk. In children exposed to doses of 1 Gy, the excess risk for TCa is equal to 7.7 [16].

Several studies conducted after the Chernobyl nuclear disaster have shown an increased incidence of TCs in subjects that at that time were aged between 5 months and 10 years [17, 18]. The average age at diagnosis of TCa was 14 years with no substantial gender-related difference in the incidence.

According to the pathology findings, the most frequent histological features were represented by solid and follicular variants of PTC. At the time of diagnosis, the disease was in an advanced stage, already exhibiting lymph node and lung metastases, a more aggressive biological behaviour, and it resulted to be more frequently associated with autoimmune thyroiditis [17, 18].

### 8.3. Previous History of Benign Thyroid Disease

In subjects suffering from benign thyroid nodules and, to a lesser percentage, in subjects suffering from goiter, a higher frequency of TC has been demonstrated [19, 20]. Such a correlation suggests either the presence of common environmental causal factors or the lack of difference in the rate of malignancy of single or multiple nodules, not yet confirmed.

Several studies reported an increased incidence of malignant nodules (from 0.4% to 9.8%) in individuals with Basedow's disease (MIM #275000) [19, 20]. These studies also noted an increased risk for those subjects who had palpable nodules, and also evaluated through neck US or thyroid scintigraphy, compared with those with diffuse non-nodular goiter. Moreover, TCs that occurred in patients with Basedow's disease seem to have a more aggressive clinical behavior [21].

Although hyperthyroidism (toxic adenoma and toxic multinodular goiter) or Hashimoto's thyroiditis (MIM #140300) do not represent additional risk factors for TCa, affected subjects have a higher risk to develop a thyroid lymphoproliferative disease such as a thyroid lymphoma [22, 23].

### 8.4. Contribution of Iodine in the Food

In areas with iodine deficiency, a higher incidence of thyroid nodules and TCs has been observed. However, after correction for the greatest number of nodules, the percentage of TCa in thyroid nodules is similar to the one found in areas with normal intake of dietary iodine. 

**Figure 2:**
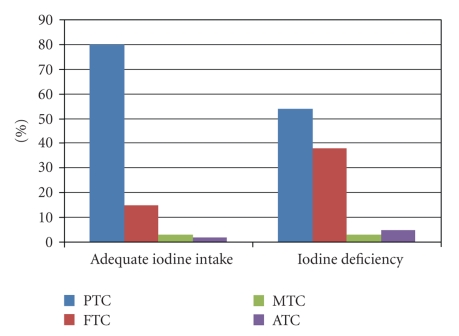
Contribution of iodine in the food to the thyroid tumorigenesis.

Different histotypes occur in accordance to the contribution of dietary iodine (Figure 2). In presence of a sufficient iodine intake, more than 80% of cancer consist of PTC; whereas in areas with iodine deficiency follicular and anaplastic figures are more frequently reported (approximately 2-3 times higher than observed in areas with adequate iodine intake) [11].

### 8.5. Body Mass Index

Several case-control studies have shown an increased risk of TCa in patients with high body mass index (BMI). The risk would be increased by 5-fold in obese men and 2 times in obese women (>97 percentile), compared to the risk observed in patients with weight <3rd percentile. In women (especially in postmenopausal age) a weight gain >14% appears to positively correlate with the onset of TCa [24, 25].

### 8.6. Hormonal Factors

The male-to-female incidence ratio has been reporting to be different according to the period of life in which TC occurs. In women of childbearing age, this ratio is about 2–4 : 1 and is reduced to 1.5 : 1 in older prepuberal and menopause individuals [26, 27]. In pregnancy, the diagnosis of goiter or thyroid nodules is frequent and an increase in thyroid volume and nodules may occur. Consequently, it has been hypothesized as a role for hormonal factors or other factors related to pregnancy in the pathogenesis of TCa [28]. However, it is still unclear why a female predominance exists [29].

## 9. Genetic Factors

In order to explain the role of genetics in thyroid carcinogenesis, we will consider the (1) presence of a positive familial history for TC and associated diseases and (2) molecular genetic aspects including (a) fine mechanisms such as gene mutations, both at nuclear and mitochondrial level, (b) gross mechanisms represented by gene rearrangements and loss of heterozygosity (LOH), and (c) a brief paragraph concerning the more recent knowledge on the role of microRNAs.

### 9.1. Familial History and Associated Diseases

In this paragraph, only nonmedullary thyroid tumors will be considered. Familial MTCs will be treated in the paragraph of *RET* proto-oncogene.

In 3%–5% of patients with TC, a positive family history for thyroid tumors in first-degree relatives can be detected. Indeed, in the setting of familial nonmedullary TCa, (FNMTCa), PTC mainly occurs in more members of the pedigree, being inherited as a dominant autosomal trait with an incomplete penetrance. In the affected subjects, the aggressiveness of the tumor is higher than that observed in the general population (high frequency of multifocal form and an higher rate of relapse compared to patients with sporadic PTC), but this observation has not been confirmed in other series [30].

Of course, genetic factors are responsible for some familial syndromes associated with high prevalence of nodular thyroid disease and TCa, such as familial polyposis of colon (FAP), Cowden's disease (CD), and Carney's complex (CNC). 

In FAP (MIM #175100), the risk of developing multicentric PTC, in particular the cribriform variant [31], is about 100 times higher than the one observed in the general population [32]. Female subjects below 35 years are more frequently affected.

In CD (MIM #158350), an autosomal dominant disease with multiple hamartomas in different tissues, the risk of PTC, or FTC is higher than observed in the general population [33].

CNC (MIM #160980) is a multiple endocrine neoplasia syndrome inherited as an autosomal dominant trait. The disease is characterized by spotty skin pigmentation, cardiac and extracardiac myxomatosis, schwannomas, and endocrine tumors, like multiple hypersecretory adrenal nodules, growth hormone-secreting pituitary tumors, gonadal tumors, and thyroid neoplasms, either follicular or papillar form. CNC is caused by loss of function mutations in *PRKAR1A* gene, which encodes the type 1A regulatory subunit of protein kinase A, a modulator of intracellular signaling of PKA (PKA; cyclic AMP (cAMP)-dependent protein kinase) [34–36].

Sporadic thyroid tumors can rarely accommodate mutations, and in fact it is very rare that these tumors can accommodate somatic mutations of *PRKAR1A* [37, 38].

### 9.2. Molecular Genetic Aspects

In recent years, the molecular bases of thyroid carcinogenesis have been thoroughly investigating. The most frequent genetic alterations, detected by molecular biology studies over the past 20 years, are mainly represented by the activation of oncogenes such as *BRAF*, *RAS*, *RET*, and *NTRK1* and the silencing of tumor suppressor genes such as *PTEN, TP53*. 

#### 9.2.1. Fine Mechanisms: Nuclear and Mitochondrial Gene Mutations

**Nuclear Genes**

***BRAF* Gene**

*BRAF* gene (OMIM #115150) encodes a protein belonging to the family of serine-threonine kinases, activator of mitogen-activated protein kinase (MAPK) with a high affinity for MEK1 and MEK2, MAP kinase kinases, leading to their phosphorylation more efficiently than other RAF isoforms [39].

MAPKs respond to mitogenic extracellular stimuli and regulate gene expression, mitosis, differentiation, proliferation, and cell survival/apoptosis. MEK1 and MEK2 activate the serine/threonine specific protein kinases ERK1 and ERK2. Activated ERKs are pleiotropic effectors of cell physiology and play an important role in the control of gene expression involved in the cell division cycle, apoptosis, cell differentiation, and cell migration [40, 41].

***BRAF* and PTC**

*BRAF* mutations are the most common genetic alterations found in PTCs, being present in approximately 45% of these tumors [42–44] (Table 2).* BRAF* mutations are present in 40%–70% of PTCs with higher percentage of positivity in more aggressive variants such as “tall cell” dedifferentiated forms [45].

In particular, the genetic alteration with the higher prevalence in classical PTCs involves the nucleotide 1799 determining a valine-glutamate substitution at amino acid residue 600 (*V600E*) with consequent activation of BRAF kinase that results in a continuous phosphorylation of MEK and MAPK pathway effectors. Such a mutation is rare in FTC [43, 46, 47].

Two more rare activating mutations of *BRAF* have been also described in PTCs: (a) the *K601E* point mutation, small in-frame insertion or deletion surrounding codon 600 and determining a lysine-glutamic substitution, and (b) the *AKAP9-BRAF* rearrangement that is more common in those PTCs associated with a previous radiation exposure [48–51]. *AKAP9* (A kinase (PRKA) anchor protein (yotiao) 9) gene encodes a member of the AKAP family, proteins which have the common function of binding to the regulatory subunit of protein kinase A (PKA) [52].

***BRAF*-PTC Genotype/Phenotype Correlation**

The characteristics of aggressiveness of PTCs, such as extrathyroidal extension, advanced presentation, presence of lymph node, or distant metastases have been associated in many studies with the presence of *BRAF* mutation [53–57], even if it has been proven to be an independent predictor of tumor recurrence, also at an early stage of disease [55, 58].

The *BRAF* mutation is thought to account for the impairment of the function of the sodium-iodine symporter (NIS) and other molecular pathways involved in the iodine metabolism of the follicular cell. In fact, *BRAF* mutation has been found to be associated either with a decreased iodine intake in some thyroid tumors or the failure of response to therapy in disease relapse [55, 59].

***BRAF* and ATC**

A *BRAF* mutation has been reported in 20% of ATCs, exhibiting also areas of well-differentiated PTC, and 15% of poorly differentiated TCs [53, 54, 60] (Table 2).

***BRAF* Mutant Animal Models**

The involvement of *BRAF* in thyroid tumorigenesis has been also suggested by studies on transgenic mice with thyroid-specific expression of *BRAF V600E* [61].

In fact, these mice developed a PTC with invasion of blood vessels, thyroid capsule, and perithyroid skeletal muscle. They are all features of aggressiveness, demonstrating a progression to poorly differentiated TC.

***RAS* Gene**

*HRAS*, *KRAS*, and *NRAS* genes are members of the *RAS* family (OMIM #109800) coding for a G-protein. When activated, RAS protein starts the intracellular signal transduction through the release of GTP and the activation of MAPK and PI3K/AKT pathways (see below). Therefore, an increase of the affinity for GTP and inactivation of the GTPase function are explained by the presence of point mutations in the *RAS* domains, especially in codons 12, 13, and 61, which determine a constantly active RAS mutant protein [62].

***RAS* and PTC**

Point mutations of *RAS* are found in 10%–20% of PTCs [63–65] (Table 2).

***RAS* and FTC/Follicular Adenomas**

*RAS* mutations have been also found in 40%–50% of FTCs and in 20%–40% of follicular adenomas, the latter with a prevalent microfollicular pattern of growth [66–70]. Often, the *NRAS* and *HRAS* mutations occur at codon 61. They have a low incidence in oncocytic tumor (designated as oncocytic if at least 75% of their constituent cells can be described as oncocytes) and, in fact, these mutations have been reported only in 0–4% of follicular adenomas and in 15%–25% of FTCs [68, 71, 72] (Table 2).

***RAS* and ATC**

Point mutations of *RAS* have been described in 18%–27% of poorly differentiated thyroid tumors and in 50%–60% of ATCs (Table 2). It is likely that mutant *RAS* facilitates a genomic instability predisposing to further genetic abnormalities as those of the *TP53* gene and then the malignant progression. An example of this relation is a case of ATC that occurred into the context of a well-differentiated FTC where in both forms a *RAS* mutation has been found, whereas a *TP53* mutation has been found only in the ATC [73].

***RAS*-PTC-FTC Genotype/Phenotype Correlation**

**PTC**

The mutation of *RAS* in PTCs is most frequently associated with the follicular variant of PTC, then to well-capsulated tumors with low rates of nodal metastases [74, 75], although some studies have found a correlation between *RAS* mutation and PTC with a more aggressive behavior as the presence of distant metastases [76].

**FTC**

In several studies, the association between *RAS *mutation and FTCs with a more aggressive behavior, presence of bone metastases, has been demonstrated. These findings support that *RAS* mutation can be associated with a less favorable prognosis of FTCs [70, 77–79].

In vitro studies showed that the mutant RAS protein could promote a chromosomal instability and then a consequent more aggressive behavior of the tumor [80, 81].

***RET *Gene**

The *RET* proto-oncogene (OMIM #164761) encodes a membrane receptor with a tyrosine kinase activity. This receptor has an extracellular domain, containing the signal peptide, the cadherin-like region and the cysteine-rich region, a single transmembrane domain, and an intra-cellular portion containing the tyrosine kinase domain [82]. The physiological ligands of *RET *belong to the glial-derived neurotrophic factors (GDNFs) family, composted by four members: neurturin, persephin, artemin, and GDNF, having a specific trophic effect on *RET *[83]. The formation of the ligand-coreceptor-receptor complex is responsible for both the activation of the kinase catalytic domain and the signal transduction which induces cells proliferation through a complex network of second messengers [84]. The tyrosine kinases are enzymes that stimulate other regulatory proteins through phosphorylation of their tyrosine residues and their subsequent activation stimulates the cell division [84].

As other members of this family, *RET* exhibits an oncogenic potential and plays a particularly important role in thyroid human cancers.

Activating chromosomal rearrangements of *RET* are involved in the tumorigenesis of some forms of PTC, and its activating point mutations account for both familial and sporadic MTC forms. In fact the 40% of PTCs are associated with somatic gene rearrangements [85].

The familial MTC is the most important clinical feature occurring within the Multiple Endocrine Neoplasia type 2 (MEN2) syndrome (OMIM #171400) [86].

MEN2 is an autosomal dominant disease described in hundreds of families throughout the World. Three distinct clinical variants of MEN2 have been reported: MEN2A, accounting for >80% of MEN2, MEN2B, and Familial Medullary Thyroid Carcinoma (FMTC). All variants of MEN2 show a high penetrance for MTC; in fact, 90% of MEN2 adult *RET* mutant gene carriers will eventually show evidence of MTC [87].

MEN2-associated *RET *germline mutations are mostly located in the cysteine-rich extracellular domain, particularly in MEN2A where they are present in 90% of cases [88–90], whereas in MEN 2B, *RET* germline mutation is predominantly at codon 918 in the intracellular tyrosine kinase domain of the protein (Table 3).

Interestingly, between 1% and 24% of individuals with MTC may have simple disease-causing germline mutations of *RET* gene [91–93] and for this reason many experts recommend DNA testing for *RET* in all patients with MTC [94].

Somatic mutations of the *RET* gene are present in 20%–80% of cases of sporadic MTCs [95, 96]. The vast majority of these mutations affect codon 918, although they have also been identified in a few other regions of the gene. Some of these somatic mutations have an heterogeneous distribution within the tumor or are detected only in a subset of metastatic nodules, thereby raising concerns that they may not be essential for carcinogenesis [95].

***RET*-MTC Genotype/Phenotype Correlation**

The reported strong correlation between genotype and clinical expression of MEN2-associated MTC have provided the opportunity to stratify three *RET* codon mutation categories of mutant carrier children [87] (Table 3).

Children with MEN2B and/or *RET* codon 883, 918, or 922 mutations are classified as having the highest risk from aggressive MTC and should be operated on within the first 6 months (Table 3).

Children with any *RET* codon 611, 618, 620, or 634 mutations are classified as intermediate level and should have thyroidectomy performed before the age of 5 years (Table 3).

Children with *RET* codon 609, 768, 790, 791, 804, and 891 mutations are classified as lower-risk level and may be operated on at a later stage (Table 3). For all groups, a more aggressive neck dissection should be performed if evidence of involved lymph nodes in the lateral neck [87] is found.

Patients with 791 mutation exhibit a lower penetrance concerning MTC o C-cell hyperplasia (CCH), suggesting that the neck surgery could be postponed up to the moment in which we assist to the increase of simulated calcitonin serum levels, underlying that some patients with this mutation will never undergo prophylactic surgery [97].

Similar findings have been obtained in patients with 649L (transmembrane domain) *RET* mutation. It has been reported that either patients carrying this mutation developed a mild MTC phenotype, thus delaying its diagnosis at an older age and confirming data already reported in literature [98, 99], or double *RET* mutants, 649L and 634, exhibited a more aggressive course, with the clinical phenotype dominated by the “more severe” mutation, C634 [96, 100, 101].

Prophylactic thyroidectomy in 649L mutant carriers should be correlated to the levels of stimulated calcitonin [101].

***N* *T* *R* *K*1 Gene**

*NTRK1* (OMIM #155240) tyrosine kinase gene is located on chromosome 1q22 and encodes for the receptor for nerve growth factor (NGF) (Table 2). Its oncogenic activation occurs through a chromosomal rearrangement [102]. *NTRK1* rearrangements are less frequent than reported for *RET* [103].

**PI3K/AKT Pathway and *PTEN* Gene Mutations**

Protein RAS and fusion protein RET/PTC may activate the PI3K/AKT (phosphatidylinositol 3-kinase/Akt) signaling pathway through the loss of function of *PTEN *[104].

PI3K are a family of related intracellular signal transducer enzymes capable of phosphorylating the inositol ring of phosphatidylinositol. AKT protein family, whose members are also called protein kinases B (PKB), plays an important role in mammalian cellular signaling.

PTEN is a protein that, following activating mutations or amplifications of the genes encoding the effector proteins of PI3K/AKT pathway, inhibits PI3K signaling. Since the PI3K/AKT pathway is fundamental in regulating cell growth, proliferation, and survival, mutations of the *PIK3CA* gene (OMIM #114480), encoding the catalytic subunit of PI3K, have been searched in thyroid tumors and found in 6%–13% of FTCs and in 0%–6% of follicular adenomas [105–107].

As mentioned above, mutations of *PTEN* gene (OMIM #601728), involved in thyroid carcinogenesis, are responsible for CD which is characterized by the occurrence of hamartomas in multiple organs, benign thyroid lesions such as multinodular goiter and thyroid adenoma and exhibit an increased risk of thyroid cancer (especially FTC) and breast [33].

***PTEN* and FTC**

Mutations of *PTEN* gene have been reported in about 7% of FTCs, whereas they have not been found in follicular adenomas [106, 107] (Table 2).

***PTEN* and ATC**

Point mutations of *PIK3CA* and *PTEN* genes have been reported in approximately 20% and 15% of ATCs cases, respectively [105, 106, 108] (Table 2).

***T* *P*53 Gene**

*TP53 *gene (OMIM #191170) encodes a protein that is essential to maintain the integrity of the genetic heritage, as it protects the body against genetic damage that induces cancer by stimulating the production of both proteins that inhibit proliferation and promote cell differentiation, either DNA repair or apoptosis.

Inactivating point mutations of *TP53* make the encoded protein unable to enter the nucleus, so it cannot longer control the production of regulating proteins, and, therefore, the above mentioned events. This has been demonstrated by studies in which the recovery of the expression of *TP53* in ATCs cultured cells would reduce the rate of proliferation, the reexpression of thyroid-specific genes (e.g., *TPO*, *PAX-8*), regaining the ability to respond to stimulation with thyroid hormone [109, 110].

Mutations of *TP53* represent the most common genetic alterations of all types of human cancers.

In the thyroid these mutations are present in approximately 60%–80% of ATCs, in 30% of poorly differentiated tumors, and only rarely in FTCs and PTCs (Table 2), mostly involving the exons 5–8 of the gene [111–115].

***C* *T* *N* *N* *B*1 (*β*-Catenin) Gene**

*CTNNB *1 gene (OMIM #116806) encodes *β*-catenin, a cytoplasmic protein which is an important intermediary in the wingless signaling pathway (WNT) [116, 117]. The Wnt signaling pathway consists of a complex network of proteins playing important roles in embryogenesis and cancer, and also involved in normal physiological processes in adult animals [118].

Point mutations at exon 3 of *CTNNB1 *gene have been found in 25% of poorly differentiated carcinomas and 66% of ATCs, respectively, but not in well-differentiated carcinoma [119, 120] (Table 2).

**Mitochondrial DNA: Gene Mutations**

Somatic point mutations and deletions of mitochondrial DNA have been found to be more frequent in adenomas and oncocytic carcinomas, whereas they are more rare in PTCs and FTCs [121].

Recently, in 15% of oncocytic tumors, but not in other types of TC, mutations in the *GRIM-19* gene (OMIM 609435), encoding for a protein involved in the process of cell death and mitochondrial metabolism, have been identified, suggesting that the alteration of *GRIM-19* gene may serve as a specific marker of such tumors [121, 122].

#### 9.2.2. Gross Mechanisms: Gene Rearrangements and Loss of Heterozygosity

**Gene/Chromosomal Rearrangements**

***RET/PTC* Genes**

In 40 % of PTCs, *RET/PTC* rearrangement has been described [85] (Table 2). The rearrangement involved the fusion between the portion 3′ of the gene for the receptor tyrosine kinase *RET* and the portion 5′ of heterologous genes [85]. *RET/PTC1, RET/PTC3*, and *RET/PTC2 *are the most frequent types of the rearrangement found in PTCs.

*RET/PTC1* and *RET/PTC3* are paracentric inversions because *RET *and *H4* (OMIM #601985) or *NCOA4* (ELE1) (OMIM 601984), the respective fusion partners, both reside on chromosome 10q where *RET* is located [123, 124].

Specifically, the abnormal fusion protein RET/PTC1(H4-RET) is a constitutively activated tyrosine kinase, whereas *NCOA4* (Nuclear receptor coactivator 4) gene encodes an androgen receptor coactivator.

On the contrary, *RET/PTC2* is due to an interchromosomal translocation between chromosome 10 and chromosome 17 [125].

Other types of *RET/PTC* rearrangements have been subsequently identified. Most of these rare types of *RET/PTC* have been found in 50%–80% of PTCs in patients with a history of previous environmental (such as the Chernobyl nuclear accident) or therapeutic exposure to ionizing radiation and in 40%–70% of PTCs of children and young adults [126–130].

Rearrangements such as *RET/ELKS* (OMIM #607127), t (10;12) (q11;p13), and *RET/HOOK3* (OMIM *607825; Homo sapiens hook homolog 3) have also been seen in patients with sporadic PTC [131, 132].

In particular, *ELKS* gene localizes onto chromosome 12p13.3 and its amino acidic sequence is rich in glutamic acid (E), leucine (L), lysine (K), and serine (S).

All the fusion events leave the domain of the receptor tyrosine kinase *RET* intact and allow *RET/PTC* oncoprotein to bind SHC (OMIM #600560), involved in RAS regulation [133]; SHC (Src homology 2 domain containing) transforming protein 1, also known as SHC1, is a human gene [134].

In approximately 20% of sporadic PTCs *RET/PTC* rearrangements have been found [135, 136]. *RET/PTC* rearrangement is regarded as an early event in thyroid carcinogenesis and it is frequently found in papillary microcarcinoma [137]. Moreover, it has been also found in adenomas and other benign lesions of the thyroid. However, since it is present in most tumor cells, it is reasonable to consider it specific for PTCs [138–140].

***P* *A* *X*8*/PPAR*γ** Genes**

PAX8/PPAR*γ* rearrangement, due to the fusion of the *PAX8* (OMIM #167415) gene with the *PPARy *gene (OMIM #601487), results from t(2;3) (q13,p25) translocation [141]. *PAX8* gene is a member of the paired box (PAX) family of transcription factors whereas *P* *P* *A* *R* *γ* gene encodes for nuclear receptor protein which functions as transcription factor regulating the expression of several genes.

PAX8/PPAR*γ* rearrangement has been found in 35% FTCs, in 2%–10% of follicular adenomas, and at a lower percentage in the Hurtle's cell carcinoma [142–144] (Table 2) suggesting these injuries to be preinvasive (in situ) forms of FTCs.

Tumors expressing such a rearrangement occur at a young age and are small in size with a solid growth pattern or nests and vascular invasion. The mechanisms of cell transformation induced by PAX8/PPAR*γ* are not fully known [143–145]. Immunohistochemical techniques allow us to detect that the rearrangement consists of an over-expression of the PPAR*γ* protein although only an intense and diffuse nuclear staining correlates with the presence of the rearrangement [141, 146].

Several studies have been conducted on this rearrangement, and various mechanisms have been observed and assumed to have a possible role on (1) inhibition of the normal function of PPAR*γ* by a dominant negative effect on the mutant protein PAX8/PPAR*γ* on PPAR*γ* wild type [141, 147], (2) activation of *P* *P* *A* *R* *γ* target genes in tumors expressing PAX8/PPAR*γ*, (3) deregulation of *PAX8* function, known to be crucial for the differentiation of thyroid cells, and (4) activation of genes not related either to the wild-type PPAR*γ* or to the PAX8 pathways [148, 149].

**LOH Studies**

Another genetic alteration, a gross alteration, reported in the TCs is LOH.

LOH represents the loss of the normal function of one allele of a gene in which the other allele was already inactivated at somatic or germline level.

LOH is detected on average in 6%–12% of follicular adenomas and in 30%–50% of FTCs.

The chromosomal regions most frequently involved are located on chromosomes 2p, 3p, 9q, 9p, 10q, 11p, 17p, and 15q [150–152].

**LOH Genotype/Phenotype Correlation**

The frequency of LOH has been correlated with the aggressiveness of the tumor and the presence of relapse in patients with FTC. In fact, in the minimally invasive tumors LOH has been detected in 30% of cases, while its frequency was greater in 50% of more aggressive cases and in the presence of disease relapse [151, 153].

A study conducted on a small group of FTCs has suggested that allelic loss of the *VHL* gene on 3p26 may serve as important diagnostic and prognostic markers of FTCs being specific for malignancy, even though its clinical usefulness should be validated in a larger group of tumors [154–157].

LOH is more present in thyroid oncocytic tumors. The most affected regions resulted to be located on chromosomes 1q, 2p, 3q, 8q, 14q, and 18q [154, 156], but also the loci on chromosome 1q and 2p showed a significantly higher rate of LOH in oncocytic carcinomas than in adenomas, with a sensitivity of 100% and a specificity of 65% for the detection of malignant tumors [156].

#### 9.2.3. MicroRNAs (miRNAs)

miRNAs are a class of small noncoding RNAs involved in a wide range of processes such as proliferation, development, apoptosis, metabolism, and response to stress. In studies conducted in different types of human tumors, it has been shown that miRNAs are abnormally expressed [158]. miRNAs profile expression analysis in human TCs, by microarray approach, detected an aberrant expression of several miRNAs in PTCs [159].

Overexpression of mir-221, -222, and-181b has been also demonstrated in transformed rat thyroid cell lines and in mouse models of thyroid carcinogenesis.

Functional studies, performed by blocking mir-221 function and by overexpressing mir-221 in human PTC-derived cell lines, have suggested a critical role of mir-221 overexpression in thyroid carcinogenesis. Taken together, these data have indicated the existence of an miRNA signature associated with PTCs, and suggested the miRNA deregulation as an important event in thyroid cell transformation [160].

Overexpression of mir-221 in PTC may drive gene expression patterns by directly and indirectly regulating numerous genes, including *HOXB5 *[161, 162]. *HOXB5* gene encodes a nuclear protein with a homeobox DNA-binding domain and the encoded protein functions as a sequence-specific transcription factor that is involved in several tissue developments [163].

**Molecular Therapy**

In oncology, understanding of the molecular mechanisms that control cell growth and survival has led to the development of new more selective and targeted pharmacological agents.

Compounds that have demonstrated a potential pre-clinical therapeutic application in TC are discussed below.

**Inhibitors of BRAF Protein**

Since alterations of BRAF are widely represented in many solid tumors, such as malignant melanoma, it is clearly appreciated the considerable interest directed to the development of specific inhibitors of BRAF activity [45, 53]. Among these, the best known is the bi-aryl urea BAY 43-9006 (sorafenib) [164]. BAY 43-9006 is an inhibitor active against RAF multikinase and other proteinkinase (VEGFR-2 and PDGFR), which can effectively block the kinase activity of BRAF [46, 165]. Preclinical studies have demonstrated its ability to inhibit the BRAF signal and growth of all of the thyroid carcinoma cell lines expressing this oncogene [166]. BAY 43-9006 has been tested on several types of human carcinomas including TCa, and the preliminary results showed a minimal or partial response in some patients [167].

Currently, numerous other compounds are being studied. They include AAL881 and LBT613, which has not demonstrated in vitro an efficacy exceeding that of sorafenib [168].

**Inhibitors of *RET***

The somatic rearrangements of the *RET* proto-oncogene, in particular those resulting from rearrangements *RET/PTC*, form various oncoproteins that represent potential molecular targets for the development of specific inhibitors. Indeed, several studies have demonstrated their high frequency in PTCs and their ability to intervene early in the process of neoplastic transformation [169].

Several inhibitors of the enzyme activity of RET have been developed, some of natural origin such as herbimicine A, clavilactones, and other synthetics [170, 171]. Their mechanism of action interferes with the ATP-binding site at the catalytic domain of RET molecule. The most effective compounds belong to the class of indolocarbazoles (CEP-701, CEP 751) [172], pyrazolopyrimidine (PP1, PP2) [173, 174], quinazoline (ZD6474) [175, 176], and indolinones (RPI-1) [177–179].

These compounds, in addition to directly inhibit the activity of RET, can also act on other downstream kinases involved by activated RET as the inhibition of Fak from PP2 [174].

ZD6474 is a tyrosinkinase inhibitor, belonging to the family of quinazoline, blocking effectively RET and the type 2 receptor of VEGF [175, 180]. In an in vitro study ZD6474 also stops the growth of human PTC cell lines expressing RET/PTC1 and prevents the growth of fibroblasts in mice expressing RET/PTC3 [181, 182].

As mentioned above, BAY 43-9006 binds to RAF-1, BRAF, and other receptor tyrosine kinases responsible for neoangiogenesis and tumor progression (VEGFR-2, VEGFR-3, FLT-3, PDGFR-B, and KIT). Moreover, BAY 43-9006 also seems to be able to inhibit the activity of RET and the tumoral growth [183].

Multikinase inhibitor, SU12248 (sunitinib), has proven to be effective in inhibiting the RET/PTC kinase signal in experimental models and it has been tested in a phase II study on differentiated TC, refractory to radiometabolic therapy and not surgically resectable [184].

EEE 788 (cetuximab) has been shown to have antiangiogenetic effects by blocking EGF-R and VEGF-R in cell lines of TC [185].

AMG706 is another multikinase inhibitor with anticancer and antiangiogenetic effects, directed selectively against VEGF receptors (VEGFR1, VEGFR2, VEGFR3), PDGF, Kit, and RET [186]. In a recent phase 1 clinical study on a small group of patients with different histotypes of TC, AMG706 seems to be well tolerated and potentially reduces either the volume of the tumor or cancer biochemical markers [187].

**Practical Applications of Molecular Genetics to the Clinical Approach for TCs**

In recent years, multiple genetic alterations have been identified in differentiated TC, leading to test the clinical utility of genetic studies. In particular, molecular genetic approaches searching for gene mutations in the material collected by FNAB may have a particular utility in small nodules and in those specimen with an indeterminate cytology.

Considering that FNA presents a false-negative rate of 1% to 11%, a false-positive rate of 1% to 8%, a sensitivity of 65% to 98%, and a specificity of 72% to 100%, its limitations are related to the skill of the operator, the expertise of the cytologist, and the difficulty in distinguishing some benign cellular adenomas from their malignant counterparts [188].

***BRAF* Mutations**

The detection of somatic *BRAF* mutations provided the diagnosis of PTC in 15%–30% of cases with doubtful cytology [189, 190]. The search for somatic *BRAF* mutations, performed on specimen obtained by FNAB, not only may allow a preoperative diagnosis, but also it is easy to be performed on small amounts of DNA and not particularly expensive since it is mainly restricted to a single mutation [191–193]. Importantly, detection of *BRAF V600E* mutation can be successfully achieved by various molecular techniques using DNA isolated from fresh or fixed FNAB samples. Four different detection methods revealed a comparable and high sensitivity of the detection in archival FNAB smears [194].

***RAS* Mutations**

The diagnostic value of searching for somatic *RAS* mutations is still controversial because it is not a specific indicator of malignancy, being also present in benign follicular adenomas. However, *RAS* mutations arise frequently in FTC and follicular variant of PTC, both histotypes difficult to diagnose at cytology performed by FNAB. Considering the key role played by mutant *RAS*, both in the progression of MTC and undifferentiating cancer, it has been recommended the surgical removal of RAS-positive adenomas in order to avoid the potential transformation into malignant forms [195].

In a prospective study aimed to analyze the role of the search for different mutations in improving the preoperative diagnosis of thyroid nodules by FNAB, the identification of *RAS* mutations has been found effective in ameliorating the diagnostic accuracy and allowing the diagnosis of malignant tumors in many samples with a previous negative or inadequate cytological diagnosis [196].

***RET/PTC* Genes Rearrangements**

The *RET/PTC *rearrangements may be sought for diagnostic purposes in the samples obtained by cytology performed with FNAB for a better definition of the preoperative diagnosis of thyroid nodules, especially in samples with indeterminate cytology or having an insufficient quantity of cells for a diagnostic purpose [140, 190, 197, 198].

**Searching for* BRAF* Mutations and *RET/PTC* Rearrangements: A Comparison among the Specificities**

When compared to the search for *BRAF* mutations, the molecular approach for *RET/PTC* rearrangements detection needs a more deep investigation since it requires the extraction of RNA and the search for at least two rearrangements of *RET (RET/PTC1* and *RET/PTC3*).

The specificity is lower because, unlike the *BRAF* mutations present only in PTCs, *RET/PTC* rearrangements may also be found in benign thyroid diseases (sporadic follicular adenomas, benign thyroid nodules, and Hashimoto's thyroiditis) [137, 139, 199–201].

***P* *A* *X*8/*P* *P* *A* *R* *γ* Genes Rearrangements**

The analysis for the *PAX8/PPARy* rearrangement may be useful in diagnosis, even if it has to be taken into account that this genetic alteration is not unique to the FTC, but it can also be found in follicular adenoma and PTC.

The identification of the *PAX8/PPARy* rearrangement needs sophisticated and expensive methods such as RT-PCR (reverse transcription-polymerase chain reaction), which is a highly sensitive technique for mRNA detection and quantitation [202], FISH (fluorescence in situ hybridization) [203], or immunohistochemistry.

It has been suggested that the detection of an intense and diffuse immunoreactivity of *P* *P* *A* *R* *γ* in tumor cells may justify the analysis of new sections of the tumor capsule and a more accurate appraisal of the suspicious areas in the search of capsular and/or vascular invasion [204].

## 10. Conclusions

TC is one of the most important malignant tumors of the endocrine system. Its incidence is increasing over the years, approximately 1% of all the new diagnoses of cancer. Its etiology appears to be multifactorial, being due to the interaction between environmental factors, among which the most important are exposure to radiation and the lack of iodine in the diet, and genetic factors. The expansion of knowledge about genetic mutations occurring in different thyroid tumors has characterized recent years, allowing the identification of a correlation between specific mutations and phenotypic characteristics of thyroid cancers, essential for their prognosis. First example is represented by BRAF mutation that appears to be an indicator of an aggressive behavior of PTC. Studies of this mutation were later extended to the development of new targeted therapies for TC such as the ones represented by inhibitors of RET- and BRAF-dependent tyrosine kinase activity, as also other molecular targets, currently under development or already in stages of clinical trial. The results of these trials should provide us with the therapeutic efficacy of these treatments and their potential use, whether developed as monotherapy or as associations of multiple drugs, especially in the treatment of aggressive thyroid tumors such as medullary carcinoma, the poorly differentiated cancer, and anaplastic carcinoma.

The application of genetic research of particular mutations to the diagnosis has allowed to improve the cytological diagnosis in those samples with FNA cytology indeterminate and/or atypical. The identification of *BRAF* mutation is particularly promising since the simple realization and the high specificity of the analysis for the determination of malignancy.

However, despite the analysis of all of the several known mutations, up to now the molecular test alone is not sufficient to detect all cases of malignancy. In fact, in various percentages in both the PTC and FTC and especially in oncocytic carcinomas, these mutations do not reach a high degree of specificity.

## Figures and Tables

**Table 1:** Correlation between incidence and histological type of TCs.

Histotype	Incidence
PTC	(i) Childhood
	(ii) <50 years
FTC	(iii) <60 years
ATC	(iv) 60–70 years

**Table 2:** Prevalence of major genetic alterations in all of the TCs but MTC.

Genes	PTC	FTC	ATC
BRAF	45%	RARE	20%
RAS	10%–20%	40%–50%	50%–60%
NTRK1	<5%	—	—
*PI3K/AKT *and *PTEN *	—	7%	15%
TP53	RARE	RARE	60%–80%
CTNNB1	RARE	RARE	66%
*RET/PTC*	25%–30%	—	—
*PAX8/PPAR*γ**	—	35%	—

**Table 3:** RET and MTC: Genotype-phenotype correlation.

RET receptor protein	*RET* mutation	CMT progression	Level of the risk of progression
Exon 10	*Cys 609*	Partially slow 5–7 years	High
	*Cys 611*		
	*Cys 618*		
	*Cys 620*		
Exon 11	*Cys 630*	Intermediated ~1 year	
	*Cys 634*		

Transmembrane domain	*RETS649L*	Slow 6–22 years	Intermediated
Exon 13	*Glu 768 Asp*		
	*Leu 790 Phe*		
	*Tyr 791 Phe*		
Exon 14	*Val 804 Met/Le*		
Exon 15	*Ser 891 Ala*		

Exon 16	*Met 918 Thr*	Fast <1 year	Very high
